# Medical Extracts

**Published:** 1884-06

**Authors:** 


### Medical Extracts.

### The action of Respirators in the treatment of Lung Diseases.

M. Moeller sums up a paper on this subject as follows:—

1. The degree of temperature of the inspired air is of no importance to the respiratory organs, whether they be healthy or diseased.

2. It is important to prevent foreign bodies, either organic or inorganic, from penetrating the respiratory organs, especially if those be diseased or threatened with disease.

This indication is particularly important with regard to persons predisposed to tuberculosis; for experience tends to establish the fact that one of the principal ways by which tubercular bacilli are introduced is through the lungs.

3. Great advantages can be derived from the inhaling of substances appropriate to the treatment of respiratory maladies, as well for the tuberculous as for those of a purely inflammatory nature.

He concludes: (*a*) That the respiratory appliances having for their object simply to raise the temperature of the inspired air are absolutely useless.

(*b*) That to render all the services one has a right to expect from those appliances they should be supplied with a layer of wadding, of sponge, or of some other substance able to arrest the inorganic particles and microscopic germs suspended in the atmosphere. In inhaling these substances through an antiseptic liquid you destroy in their passage the microbes suspended in the inspired air, and at the same time act on the diseased respiratory organs; but it is necessary that the inhaler be supplied with valves so arranged that the air exhaled does not follow the same route as the air inhaled.

(*c*) The apparatus of Dr. Graham Brown, of Edinburgh, offers this advantage, that it employs the heat of the exhaled air to raise the temperature of the wadding and of the medicated liquid, which increases the activity of the antiseptic emanations. For this reason this inhaler will be preferable whenever a therapeutic effect is desired.—*Bulletin de l'Académie Royale de Médecine de Belgique*, Tome xvij., No. 11, 1883.

### On the changes in the position of the Brain with different attitudes of the Body.

M. J. Luys believes that he has been able to establish the following facts :—

1. The brain is not immovably fixed in the cranial box which surrounds it, as is commonly believed. Over and above the intrinsic movements of expansion and diminution, which are admitted by all, it is endowed with certain proper movements independent of the preceding ones.

2. When the human subject passes successively from the dorsal to the abdominal decubitus the brain follows the movement, and glides evenly from behind forwards simply by the action of gravity, owing to the smooth condition of the serous arachnoid.

3. When the body assumes the vertical position the cerebral mass presses down on itself, and in breaking contact with the parietes of the cranium leaves an empty space, which may be estimated at about six millimetres in thickness in the dead subject, and probably less in the living.

4. When the body is in the lateral decubitus the cerebral mass still obeys the laws of gravitation ; the upper lobe presses lightly on its fellow through the falx cerebri, which gives only a partial support.

The material fact of the motility of the brain being admitted, he reviews the various physiological and pathological questions which depend upon it. The mechanical difficulties in the cerebral circulation through the circle of Willis when the body is in a vertical position ; the production of mal-de-mer ; various forms of headache ; nocturnal epilepsy ; some forms of heat-fever ; and various symptoms which manifest themselves when the body is in a horizontal position, are believed to be satisfactorily explained by the mechanical alteration in the position of the brain. The author further adds a caution, that invalids whose brains are sensitive, or who are the subjects of chronic brain lesions, should abstain as much as possible from travels of all kinds, more especially by rail, which is a potent cause of brain exhaustion.—*Bulletin de l'Académie de Médecine*, 2me. Série, Tome xiij., March, 1884, No. 13.

### On a Surgical treatment of Sciatica.

Dr. Comingor, in *The American Practitioner* for February, 1884, relates some striking cases of cure of sciatica by immobilization of the limb in plaster-of-Paris bandage.

The patient is put under chloroform; the limb is put through its normal movements; the soft structures are stretched and massaged; and the bandage is then applied to the whole limb, from the foot upwards, and encircles the pelvis. The patient is permitted to get up on crutches, and the bandage is removed in a fortnight or three weeks. Relief is usually immediate and permanent.

### Malignant Pustule communicated by a Fly.

The case was under the care of M. Mollière at the Hôtel Dieu at Lyons.

The patient was bitten on the cheek by a large black fly. In a few hours it began to itch, and next day swelling set in. When admitted to hospital the patient's whole cheek was enormously swollen, and livid in colour. In the centre of the swelling was a small black phlyctena, surrounded by a number of transparent vesicles. There was no fever and no constitutional symptom.

M. Mollière at once destroyed the pustule with the thermo-cautery, and then freely injected the surrounding parts with a 20-per-cent solution of carbolic acid. The affected surface sloughed off in a week, and in three weeks the patient was discharged.

An animal inoculated with the fluids died with all the signs of specific gangrenous infection.—*Gazette des Hôpitaux*, No. 102.

### Excision of the Uvula.

M. Tholozan reports, in a communication presented to the Academy of Medicine in Paris, on January the 8th, 1884, that in the Districts of Semnan and Firouz-Kouh, near to Téhéran, excision of the uvula is practised by the Persian barbers on newly-born children as a prophylactic against inflammation of the throat. The custom appears to be an ancient one, dating from at least five or six generations, and limited to two small towns and the surrounding villages. The barbers had never seen any danger or accident arising from the practice, and the inhabitants declared that they had never heard of any accident therefrom. They believed that in this way the children are protected from the danger of suffocation in various diseases of the throat, and that attacks of these maladies are thereby rendered less frequent and less severe.—*Bulletin de l'Académie de Médecine*, 2me. Série, Tome xiij., No. 1.

### Physiological Action of Kairin.

To the Société de Biologie, Paris, M. Loych communicates the results of his experiments upon the physiological action of kairin, which he had made in conjunction with M. Brouardel. In his investigations of this substance, M. Hallopeau has not insisted sufficiently on examination into the state of the blood. Under its use, complete cyanosis of the tongue and lips is observed, and after death the lungs are found shrunken, the spleen blackish, &c. It was therefore important to ascertain the action of kairin on the blood.

On examining the gases contained in the blood, there was found, before the administration of kairin, in each 100 c.c. of blood 30 parts of carbonic acid, 14 of oxygen, and 1.5 of nitrogen; after injection of kairin in the same animal, there were 21.9 of carbonic acid, 3.6 of oxygen, and 1.5 of nitrogen. A considerable diminution of the respiratory capacity of the blood occurs therefore from the administration of kairin. From this must be inferred a destruction of hæmoglobin as the explanation of the lowering of the temperature, of the hæmaturia, cyanosis of lips, anæsthesia, and so forth. Its employment would be counter-indicated in typhoid fever, scarlatina, phthisis, &c., all maladies in which it is desirable to seek to increase rather than diminish the oxy-hæmoglobin of the blood.—*Gazette des Hôpitaux*, May 6th, 1884.

### Fournier on Hereditary Cerebral Syphilis.

Dr. Fournier, at the Hôpital St. Louis, in a course of lectures calls attention to the cerebral complications of late hereditary syphilis, premising two general ideas: 1st, hereditary cerebral syphilis reproduces almost all the symptoms which are met with in the adult from acquired syphilis; and 2nd, the hereditary form in adolescence, like the acquired in the adult, manifests its commencement in very different ways, but like that also tends to an uniform ending—loss of power, both intellectual and motor. Various though these early symptoms may be, there are some which occur first, and must be noticed specially. Three sets of symptoms, singly or combined, form the beginning of the malady: 1st, convulsive symptoms; 2nd, headaches; 3rd, affections of the intellect.

1st. To treat of the epileptic symptoms first. On account probably of the predisposition of children to convulsive attacks, epileptiform convulsions are of very frequent occurrence in the cerebral syphilis of childhood, and, indeed, are found in all stages of cerebral syphilis of childhood and adolescence. As an initial symptom it is found in two forms: epilepsy pure and simple, and epilepsy complicated with other cerebral phenomena. The first form is very rare, and presents no features to distinguish it from essential epilepsy. In the complicated form, the most common additional symptoms are, frequent intermittent headaches, noises in the head, vertigo, dimness of sight, dizziness, alteration of character, weakening of the intellect, and various combinations of these symptoms. These initial symptoms may be continued also in two forms: they may retain the purely epileptic form for a greater or less time, without the addition of other phenomena of a different kind; or the initial epilepsy, whether complicated at first or not, is lost in the symptoms of a coarse lesion of the brain.

In the first group, for months or years the patient seems to be the subject of a pure epilepsy only; but after a long time the supervention of other symptoms shows that it is indeed a symptomatic epilepsy. These cases are rare, and are naturally most liable to be mistaken as regards both diagnosis and treatment; indeed, it is only the results of treatment which absolutely prove their true nature.

In the second group, the epileptiform attacks at first predominant subside to the position of being one of many factors. These other factors, as already stated, may be congestive troubles, as tinnitus aurium; moral troubles, as irritability; intellectual troubles, as loss of memory; or paralytic troubles, as hemiplegia. Whence we see that hereditary cerebral syphilis of childhood observes the same succession of symptoms as in the adult.

In the treatment of this form of cerebral syphilis, we may hope for all or nothing, according to the case. The two principal factors which determine the result of treatment are: first, the early or late commencement of specific treatment; second, the exclusively epileptic character of the symptoms, or the presence also of other grave cerebral symptoms. Cure is almost certain when treatment is begun early, at a period when epilepsy exists alone, or when at least there are but slight other cerebral symptoms, the epileptic form being the most amenable to treatment. On the other hand, failure is almost certain when the epilepsy is of long standing, and especially when other grave cerebral symptoms co-exist, such as weakness of intellect, general muscular paresis, sensory troubles, impairment of speech; such cases are well-nigh beyond the resources of art. The fact of the epilepsy having existed for some months is not of so serious a prognosis; certain cases of months' or even years' standing are still susceptible of cure if not complicated by other cerebral symptoms. The essential point, therefore, for practice is, to make, as early as possible, a correct diagnosis of the nature of the pseudo-epilepsy, and treat it by the only kind of remedies which are applicable to it. This is done by investigating closely into the antecedents of the patient and his family, since the nerve-phenomena depending upon syphilis do not differ from those depending upon non-specific causes. The patient must be minutely examined for signs of hereditary syphilis; if none should be discovered, then the parents must also be examined, and all the brothers and sisters of the patient as well; for, as Dr. Hughlings Jackson points out, the marks of hereditary syphilis may be found in some of the children younger than the patient, as well as more probably in those older.

Headaches and Affections of the Intellect in subsequent lectures.—*L'Union Médicale*, Nos. 62 *et seq.*

### The Treatment of Burns.

Thanks to the antiseptic method of wound-dressing, it is now possible to do away with almost all of the disagreeable features which were formerly connected with the treatment of an extensive burn; and such a case may be made to arise from the insignificance of a nuisance and to assume the dignity of an object for enthusiastic interest. Frequent dressing is not only uncalled for, but is very injurious. Odors need not be produced; and as for suppuration, that may be relegated to the past as a pathological curiosity. Nature need not become discouraged and refuse to help the timorous epithelium cells across the little stepping-stones of her granulating surfaces; and repair may be made to go on so rapidly and with such vigour that lagging seldom occurs.

Burns of the first degree, in which the skin is hyperæmic, but is not destroyed at all, are usually of not much importance; but the stinging, burning pain always calls for relief, and this may be promptly and completely relieved by the following method: 1. Tear any convenient soft fabric into strips a couple of inches wide, and spread them thickly with a mixture of carbonate of lead and vaseline in equal parts. 2. After the strips of painted cloth have been applied smoothly over the burned surface, cover the whole with a piece of gutta-percha tissue or oiled silk. 3. Cause a free movement of the bowels. If the carbonate of lead is mixed with vaseline there is no danger of absorption; but if any animal or vegetable oil should be used, there might be some risk in applying this dressing. I have, however, used ordinary white paint on several occasions without getting any symptoms of lead-poisoning. The gutta-percha tissue prevents the dressing from drying, and adds an element of neatness which is quite important.

If a small portion of the body have been burned, as, for instance, the forearm and hand, the plan to be carried out would be as follows: 1. Anæsthetize the patient. 2. Pull off all of the cuticle which is loose, and all that has been raised in blebs and vesicles. 3. Lay the arm on a towel which has been wrung out in bichloride of mercury solution (1 to 2,000), and carry a rubber blanket underneath all; arrange the rubber blanket in such a way that irrigating fluids shall run into a pail placed for their reception. 4. Scrub the burned area and the skin in its vicinity very thoroughly with a soft brush, and at the same time bathe the parts copiously with bichloride of mercury solution (1 to 2,000) or with a solution of salicylic and boracic acids in the proportion of one grain of the former and six grains of the latter to the fluid ounce of water. 5. Cover the burned surface evenly with strips of protective oiled silk, which have been stored in an antiseptic solution. 6. Sprinkle iodoform along the margins of the strips of protective. 7. Place several layers of carbolized or sublimated gauze over the protective, and cover still further with a thick wadding of borated cotton placed between layers of antiseptic gauze. 8. Apply snugly a carbolized roller bandage. 9. Keep the bowels open. 10. Quiet constitutional disturbance with bromide of potassium and chloral hydrate. The dressing should not be disturbed until the eighth day; and when it is removed it will be found that everything is completely healed, and no further treatment is necessary. Of course, the brush which we use has been washed in an antiseptic solution, and the surgeon's hands must be most carefully prepared before he touches the case.

In another class of cases, where a very large surface of the body has been burned to the second degree, we shall often find it impossible to apply the thoroughly antiseptic dressing which has just been described; and the subnitrate of bismuth treatment, which stands next in value, should be applied as follows: 1. Anæsthetize the patient with chloroform. 2. Remove all clothing, and whatever may adhere to the burned surfaces. 3. Wash all of the injured area with an antiseptic solution. 4. Pull away all loose cuticle, and as fast as it is removed sprinkle the parts beneath thickly with subnitrate of bismuth. 5. Cover lightly with a single layer of soft cloth or sheet-lint. 6. Remove the cloth covering once or twice daily; and wherever any of the subnitrate of bismuth has been loosened by the discharge, sprinkle more of the powder on the place. 7. During the period of depression and congestion, sustain the heart and relieve the shock of the nervous system by the use of hypodermic injections of morphine. 8. During the period of inflammation, support the heart and aid the inflamed kidneys with digitalis. Quiet the disturbed stomach with belladonna, and give refreshment in the shape of acid drinks. Feed the patient by the rectum, and use peptonized milk only for this purpose. 9. During the period of reaction continue feeding by the rectum, and for the first time cause a free movement from the bowels, using a saline cathartic. 10. When reaction is well established, commence to stimulate the patient with sherry wine, and gradually coax the stomach to bear light, varied diet.—Dr. ROBERT T. MORRIS, in *The New York Medical Record*, May 17th, 1884.

### Dr. S. C. Gordon on the Treatment of Gonorrhœa by the Hot-water Douche.

The writer fills a fountain-syringe with a quart of water at a temperature of 100° F., and introducing the tube as high as he can push it, he injects it and allows the fluid to run out. If this gives relief he increases the temperature of the water and repeats the injection, doing this twice or thrice daily. He thus summarises his results:—

"1. I should hope, and confidently expect, to abort, in from three to five days, a large majority of cases that were treated as soon as the first well-known symptoms appeared. In these cases I use the injection as *hot as it can possibly be borne*—three or four times in twenty-four hours; at least two quarts should be used at each time.

"2. In cases of ten days' or two weeks' duration (at which time the inflammatory process has ended), I believe the most of the trouble can be relieved in a very few days. The suffering so characteristic of that stage will usually pass away in twenty-four hours. In many of these cases I have been enabled to force the water into the bladder, and then allow the patient to pass it away immediately. This has a good effect upon the dysuria, relieving it almost at once. In this class of cases, also, the water should be very hot.

"3. Where two or three days have elapsed before the patient is seen, I do not expect so much as in the former classes. Even here much may be done by the external use of very hot water, and the careful, gentle use of the fountain-syringe, filled with *simple warm water*, at about the temperature of the body, or less. This promotes cleanliness, and is a sedative."—*The New York Medical Journal*, April 19th, 1884.

### Surgical Delusions.

*Chloroform Anæsthesia.*—Many still cling to the delusion that chloroform is a safe anæsthetic, because they have never seen a patient die from it. Is one man's experience to weigh against the physiological, the experimental, the clinical experience of the whole world? Dare we employ chloroform instead of ether, when recognized authorities state that in chloroform anæsthesia death occurs without warning in the hands of experienced administrators, when some five hundred deaths have already been reported, when Schiff and Dalton reject it in physiological laboratories because of its mortality, when the Scientific Grants Committee of the British Medical Association assert that chloroform is a more dangerous anæsthetic than ether? Adherence to chloroform in the face of such facts is criminal, when circumstances permit ether to be obtained. The assertion that it is often impossible to produce anæsthesia with ether is the result of inefficient methods of administration. Ether, if given as chloroform is and should be given, is in truth a useless anæsthetic, but given properly it is efficient.

*Value of Styptics.*—The belief in the necessity of styptics is a delusion less dangerous than that just mentioned, but is given more extended credence. Such agents are seldom, probably never needed in general surgery to arrest hemorrhage. When ligatures, torsion, or acupressure is not demanded (and such is seldom the case unless the artery is as large as the facial), moderate direct pressure applied in dressing the wound is the only hemostatic required. Styptics often do harm, and, as they are not needed, they should be discarded.

*Fatality of Small Hemorrhages.*—There is much misapprehension about the quantity of blood that a healthy man can lose with impunity. Many who often look with equanimity upon a parturient woman losing a pint of blood from the uterine sinuses, would be dismayed at a man losing half or quarter that amount during removal of a tumor. While not advocating needless waste of blood, and especially in patients suffering surgical shock, I assert that there is an unnecessary fear of blood spurting from a few insignificant vessels. The largest artery can be controlled by pressure not greater than is used for ringing the electric bell in your hotel. Hence there is always sufficent power in your fingers to obviate hemorrhage until strings can be obtained and applied.—Dr. JOHN ROBERTSON, in *The Medical and Surgical Reporter*, Philadelphia, May 24th, 1884.

